# New mutations in the *PKD1 *gene in Czech population with autosomal dominant polycystic kidney disease

**DOI:** 10.1186/1471-2350-10-78

**Published:** 2009-08-17

**Authors:** Jitka Stekrova, Jana Reiterova, Stanislava Svobodova, Vera Kebrdlova, Petr Lnenicka, Miroslav Merta, Ondrej Viklicky, Milada Kohoutova

**Affiliations:** 1 Institute of Biology and Medical Genetics of the 1st Faculty of Medicine and General Teaching Hospital, Charles University, Albertov 4, Prague 2, 128 00, Czech Republic; 2 Department of Nephrology of the 1st Faculty of Medicine and General Teaching Hospital, Charles University, U Nemocnice 2, Prague 2, 128 00, Czech Republic; 3 Institute of Clinical and Experimental Medicine, Videnska 1958, Prague 4, 140 00, Czech Republic

## Abstract

**Background:**

Autosomal dominant polycystic kidney disease (ADPKD) is the most common hereditary renal disease. The disease is caused by mutations of the *PKD1 *(affecting roughly 85% of ADPKD patients) and *PKD2 *(affecting roughly 14% of ADPKD patients) genes, although in several ADPKD families, the *PKD1 *and/or *PKD2 *linkage was not found. Mutation analysis of the *PKD1 *gene is complicated by the presence of highly homologous genomic duplications of the first two thirds of the gene.

**Methods:**

The direct detection of mutations in the non-duplicated region of the *PKD1 *gene was performed in 90 unrelated individuals, consisting of 58 patients with end-stage renal failure (manifesting before their 50^th ^year of life) and 32 individuals from families where the disease was clearly linked to the *PKD1 *gene. Mutation screening was performed using denaturing gradient gel electrophoresis (DGGE). DNA fragments showing an aberrant electrophoretic banding pattern were sequenced.

**Results:**

In the non-duplicated region of the *PKD1 *gene, 19 different likely pathogenic germline sequence changes were identified in 19 unrelated families/individuals. Fifteen likely pathogenic sequence changes are unique for the Czech population. The following probable mutations were identified: 9 nonsense mutations, 6 likely pathogenic missense mutations, 2 frameshifting mutations, one in-frame deletion and probable splice site mutation. In the non-duplicated region of the *PKD1 *gene, 16 different polymorphisms or unclassified variants were detected.

**Conclusion:**

Twenty probable mutations of the *PKD1 *gene in 90 Czech individuals (fifteen new probable mutations) were detected. The establishment of localization and the type of causal mutations and their genotype - phenotype correlation in ADPKD families will improve DNA diagnosis and could help in the assessment of the clinical prognosis of ADPKD patients.

## Background

ADPKD is the most frequently inherited renal cystic disorder with an incidence between 1 in 400 to 1 in 1000 [1]. ADPKD is a systemic disorder with cysts and connective tissue abnormalities involving many organs [2]. The progressive formation and enlargement of renal cysts causes the decline in renal function.

The disease is genetically heterogeneous. At least three different genes are involved. The *PKD1 *locus (MIM 601313) is linked to the short arm of chromosome 16, at 16p13.3 [3,4]; and so far, 818 different sequence variants have been reported in Polycystic Kidney Disease Mutation Database (PKDB) [5]. Later, a second locus *PKD2 *(MIM 173910) was located on chromosome 4q21 [6]; and 123 different sequence variants have been reported in PKDB [5]. Families with ADPKD unlinked to the *PKD1 *or *PKD2 *loci have also been described [7], but the predicted *PKD3 *locus (MIM 600666) has not been mapped yet. Eighty-five percent of ADPKD cases are caused by a mutation of the *PKD1 *gene on chromosome 16 and in about 15% of all cases, the *PKD2 *gene on chromosome 4 is mutated [8]. When PKD2 patients are compared to PKD1 patients, they seem to have a milder clinical presentation. The average age of PKD1 patients at end-stage renal disease (ESRD) is 54.3 years compared with 74.0 years for PKD2 patients [9].

The *PKD1 *gene consists of 46 exons from which a 14.1 kb transcript is produced. The 4303-aminoacid product of *PKD1 *is called polycystin-1. The *PKD2 *gene consists of 15 exons. The 5.4 kb *PKD2 *transcript encodes the protein polycystin-2, which consists of 968 amino acids. Polycystin-2 is a TRP (transient receptor potential) ion channel, which is involved in regulation of intracellular Ca^2+ ^concentration [10]. The cytoplasmic COOH terminus of polycystin-2 interacts with the coiled-coil region of polycystin-1 [11,12]. Both polycystins, similarly to other cystogenic proteins, have been localized to primary cilia [13]. This complex may act as a flow-dependent mechanosensor that regulates the differentiated state of tubular epithelial cells [14].

ADPKD is the disease with variable clinical course not only among families with different mutations, but within families with a defined mutation as well. Different mutations in *PKD *genes may have different effects on the clinical course of the disease. The genotype-phenotype correlation of 80 families revealed earlier ESRD (49 years versus 54 years) for mutations in the 5'region of the *PKD1 *gene (to exon 19, nucleotide 7812) in comparison with mutations in the 3'region [15]. Very early onset of the ESRD has been described in families with mutations in exons 15 and 41 of the *PKD1 *gene [16,17]. As for *PKD2 *patients, those mutations at the 3'end of the *PKD2 *gene result in a milder clinical course; however there is no clear correlation between the age of onset of ESRD and the location or the type of mutation [18]. The majority of *PKD1 *and *PKD2 *mutations are unique to a single family, recurrent mutations account only for 30% of the total. Detection levels are from 60 to 70% [19]. In this study, the non-duplicated region of the *PKD1 *gene in 90 unrelated Czech individuals was screened.

## Methods

### Patients

The coding sequences of the non-duplicated region of the *PKD1 *gene in 90 unrelated Czech patients with ADPKD were screened. The study was performed with the approval of the Ethics Committee of the General Teaching Hospital in Prague. Informed consent was obtained from each patient before genetic testing. Fifty-eight ADPKD patients (hemodialysis-HD 1-58) from dialysis centres in the Czech Republic, from the Transplantation Centre in Prague and from the Department of Nephrology of the General University Hospital in Prague were selected. This group (34 males, 24 females) that reached ESRD were all aged between 33 and 50 (average age at onset of ESRD is 44.5 ± 4.8 years). Other 32 unrelated patients (18 males, 14 females) with ADPKD clearly linked to the *PKD1 *gene, according to the linkage analysis in Czech families, were selected (data not shown).

Asymptomatic at-risk individuals were examined by ultrasound. The positive diagnosis of ADPKD was based upon the criteria described by Ravine [20]. In most patients, the onset of arterial hypertension was established retrospectively. Hypertension was defined as a blood pressure higher than 140/90 mmHg (repeatedly measured) or a normal blood pressure maintained by the use of antihypertensive drugs.

### Mutational screening

**Figure 1:**
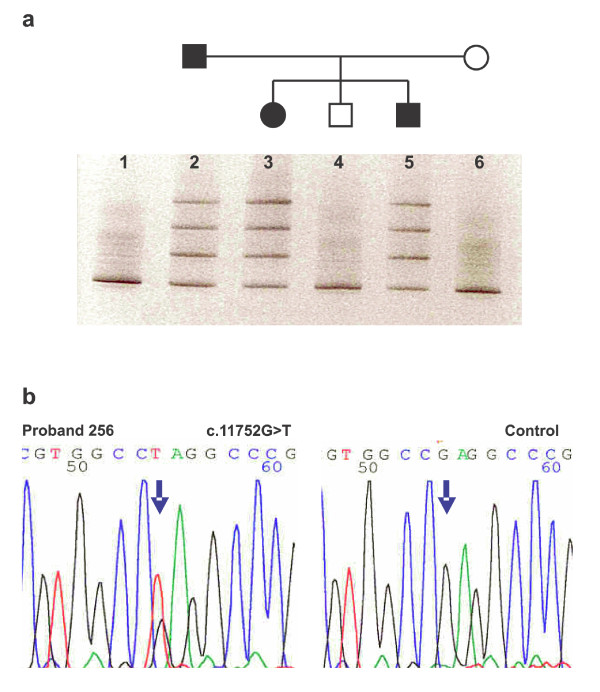
**The segregation of the *PKD1 *germline mutation p.E3918X with the disease in family 256**. **a**. Denaturing Gradient Gel Electrophoresis of PCR products of part A of exon 43 of the family 256. Lane 1 represents control. Lanes 2, 3 and 5 represent heterozygous affected members of the family. Lanes 4 and 6 represent healthy members of the family. **b**. Sequencing pattern of part of the exon 43 (forward) in proband of the family 256 with heterozygous substitution G>T at nucleotide position 11752 of the *PKD1 *gene (p.E3918X) and control.

Genomic DNA was isolated from peripheral blood lymphocytes by the salting-out procedure [21]. The coding region and intron-exon boundaries of the non-duplicated region of the *PKD1 *gene were screened for mutations using Denaturing Gradient Gel Electrophoresis (DGGE). Exons 34 - 46 were amplified using the set of GC-clamped primers. Sequences of primers, PCR and DGGE conditions are described in detail by Perrichot *et al*. [22]. All PCR fragments showing an aberrant electrophoretic banding pattern on DGGE were sequenced in both directions using an automatic fluorescent genetic analyzer (ABI Prism™ 310 Genetic Analyzer; PE Applied Biosystems) in accordance with the manufacturer's instructions. To prove the segregation of the potential mutation with the disease in the family, the DNAs of all available members of the concrete family were analyzed using DGGE (Figure 1a) and/or sequencing.

In the case of not-yet described missense mutations, 200 healthy individuals were tested for the presence of this potential mutation. This test was performed by restriction analysis (in case that the restriction site is created or abolished as a consequence of the found sequence change), high resolution melting analysis (HRM; LightCycler^® ^480 Roche instrumentation) or SNaPshot™ Multiplex Kit (ABI PRISM^310 ^instrumentation) according to the manufacturer's instructions.

### Data analysis

**Figure 2:**
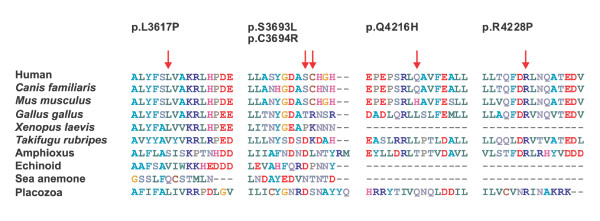
**The graphic alignment of putative causal missense mutations in *PKD1 *gene**. Multiple sequence alignment of protein sequences was performed with distant species: Human - sp|P98161-3|PKD1_HUMAN Isoform 3 of Polycystin-1; *Canis lupus familiaris *- ref|NP_001006651.1| polycystin 1; *Mus musculus *- ref|NP_038658.2| polycystin 1; *Gallus gallus *- ref|XP_414854.2| PREDICTED: similar to polycystin 1; *Xenopus laevis *- gb|AAT77543.1| polycystic kidney disease protein 1 precursor; *Takifugu rubripes *- gb|AAB86683.1| unknown; Amphioxus - *Branchiostoma floridae *ref|XP_002202413.1| hypothetical protein BRAFLDRAFT_64810; Echinoid - *Strongylocentrotus purpuratus *ref|XP_001194831.1| PREDICTED: similar to receptor for egg jelly 4;Sea anemone - *Nematostella vectensis *ref|XP_001640030.1| predicted protein; Placozoa - *Trichoplax adhaerens *ref|XP_002110052.1| hypothetical protein TRIADDRAFT_53596. The positions of putative missense mutations are marked by red arrows.

Putative missense changes in *PKD1 *gene were analyzed for their site conservation in protein sequences BLAST [23]. Multiple sequence alignment of protein sequences and the graphic view of the alignment were performed using BioEdit Sequence Alignment Editor (Figure 2) [24]. The position of a resultant amino acid change within polycystin-1 was determined at UniProtKB/Swiss-Prot entry P98161[25].

## Results and discussion

Twenty (19 different) germline likely pathogenic sequence changes in 90 unrelated patients were identified by mutation analysis of the coding non-duplicated region of the *PKD1 *gene (Table 1). In one individual, two different likely pathogenic sequence changes were detected. Overall detection rate was 21.1% (19 out of 90 unrelated Czech patients), which is relatively high level considering that only non-duplicated region of the gene was screened. The high detection level of putative mutations confirms that DGGE is a reliable and sensitive (but time consuming) method for screening of mutations. It allows the resolution of relatively large DNA fragments, up to 500 bp. It is also capable to detect up to 100% of all single base substitutions. It has been shown to be more sensitive than many other commonly used mutation detection methods, including single strand conformation polymorphism (SSCP) and heteroduplex analysis [26]. DGGE has a number of limitations, including the use of specialized equipment, the addition of GC clamps (that makes the purchase of PCR primers expensive) and the knowledge of melting behavior for an efficient analysis.

**Table 1:** Likely pathogenic sequence changes identified in the non-duplicated region of the *PKD1 *gene in Czech patients with ADPKD

**Family**	**Exon/Intron**	**Nucleotide change**	**Aminoacid change/** **Predicted effect**	**Reference**
**HD 53**	**34**	**c.10462 C>T**	**p.Q3488X**	**Current paper**

**HD 15**	**IVS36**	**c.10821+1G>C**	**probably splice defect**	**Current paper**

**340**	**37**	**c.10850 T>C**	**p.L3617P **(Fig. 2)	**Current paper**

**173**	**38**	**c.11078 C>T**	**p.S3693L **(Fig. 2)	**Current paper**

**HD 15**	**38**	**c.11080 T>C**	**p.C3694R **(Fig. 2)	**Current paper**

**329**	39	c.11258 G>A	p.R3753Q	[19]

**HD 2**	**40**	**c.11340_11345 delTTACGA**	**p.Y3781_D3782del **(Fig. 3a)	**Current paper**

**HD 12**	**40**	**c.11345dup8**	**p.D3782EfsX46 **(Fig. 3b)	**Current paper**

**HD 23**	**41**	**c.11417 G>A**	**p.W3806X**	**Current paper**

**HD 41**	**42**	**c.11623_11645del22**	**p.A3875PfsX62**	**Current paper**

**256**	**43**	**c.11752 G>T**	**p.E3918X **(Fig. 1)	**Current paper**

**HD 10**	44	c.12031 C>T	p.Q4011X	[29]

**323**	**44**	**c.12056 T>G**	**p.L4019X**	**Current paper**

**281**	44	c.12061 C>T	p.R4021X	[27]

**HD 31**	44	c.12061 C>T	p.R4021X	[27]

**387**	44	c.12124 C>T	p.Q4042X	[27]

**237**	**46**	**c.12648 A>C**	**p.Q4216H **(Fig. 2)	**Current paper**

**HD 38**	**46**	**c.12683 G>C**	**p.R4228P **(Fig. 2)	**Current paper**

**HD 1**	**46**	**c.12691 C>T**	**p.Q4231X**	**Current paper**

**HD 40**	**46**	**c.12724 C>T**	**p.Q4242X**	**Current paper**

Novel probable mutations in **boldface **type. HD - patients from dialysis centres in Czech Republic, IVS - the intronic sequence; Current paper - mutation was not described in The Polycystic Kidney Disease Mutation Database (PKDB server) [5] and/or in The Human Gene Mutation Database at the Institute of medical Genetics in Cardiff (HGMD^® ^server) [30].

Fifteen new likely pathogenic sequence changes in Czech population were identified. Twelve likely pathogenic sequence changes in 11 patients from 58 individuals (19.0%), who had reached ESRD before the age of 50 years, were found. Eight putative mutations in 32 families (25.0%) with clear linkage to the *PKD1 *gene were identified. Ten nonsense mutations (50.0%), six likely missense mutations (30.0%), two frameshifting mutations (10.0%), one in-frame deletion (5.0%) and one putative splice site mutation (5.0%) were identified in total. In the non-duplicated region of the *PKD1 *gene, sixteen different polymorphisms were also described (Table 2).

**Table 2:** Polymorphisms identified in the non-duplicated region of the *PKD1 *gene in Czech patients with ADPKD

**Allele frequency**	**Exon/Intron**	**Nucleotide change**	**Aminoacid change/** **Predicted effect**	**Reference**
3.3%	35	c.10529 C>T	p.T3510M	[31]

20.0%	35	c.10535 C>T	p.A3512V	[32]

4.4%	36	c.10768 C>T	p.L3590L	[33]

1.1%	IVS38	c.11156+13 G>A	Likely silent	[22]

1.1%	40	c.11346 C>T	p.A3782A (Fig. 3c)	[34]

2.2%	IVS41	c.11537+5_+6insGGG	Likely silent	[35]

2.2%	IVS43	c.12004-34 C>A	Likely silent	[29]

17.8%	44	c.12133 A>G	p.I4045V	[36]

3.3%	IVS44	c.12138+22delG	Likely silent	[37]

8.9%	45	c.12176 C>T	p.A4059V	[36]

1.1%	45	c.12270 C>G	p.L4090L	[38]

12.2%	45	c.12276 A>G	p.A4092A	[39]

10.0%	45	c.12409 C>T	p.L4137L	[39]

1.1%	45	c.12436 G>A	p.V4146I	[39]

17.8%	46	c.12630 T>C	p.P4210P	[40]

**2.2%**	**46**	**c.12666 C>T**	**p.L4222L**	**Current pap**.

cDNA numbering is based on reference database: The Polycystic Kidney Disease Mutation Database (PKDB server) [5].

Novel polymorphisms in boldface type. IVS - the intronic sequence; Current paper - mutation was not described in The Polycystic Kidney Disease Mutation Database (PKDB server) [5] and/or in The Human Gene Mutation Database at the Institute of medical Genetics in Cardiff (HGMD^® ^server) [30].

Clinical characteristics of patients/probands with an identified putative mutation are summarized in Table 3.

**Table 3:** Clinical characteristics of patients/probands with identified likely pathogenic mutation

**Patient/Proband**	**Likely pathogenic** **sequence changes**	**Gender** **F/M**	**Age of ESRD (years)** **Creatinine (μmol/l)**	**HT** **Y/N**
HD 53	p.Q3488X	F	48	Y

HD 15	Splice (c.10821+1G>C) and p.C3694R	F	45	Y

Fam.340	p.L3617P	F	52 y- creat. 350	N

Fam.173	p.S3693L	F	55	Y

Fam.329	p.R3753Q	F	58 y- creat. 230	N

HD 2	p.Y3781_D3782del	M	42	Y

HD 12	p.D3782EfsX46	M	45	Y

HD 23	p.W3806X	F	42	Y

HD 41	p.A3875PfsX62	F	44	Y

Fam. 256	p.E3918X	F	50	N

HD 10	p.Q4011X	F	48	Y

Fam. 323	p.L4019X	M	50 y- creat. 104	Y

Fam. 281	p.R4021X	F	50	Y

HD 31	p.R4021X	M	36	Y

Fam.387	p.Q4042X	M	32 y- creat. 154	Y

Fam. 237	p.Q4216H	F	42	Y

HD 38	p.R4228P	M	47	Y

HD 1	p.Q4231X	F	50	Y

HD 40	p.Q4242X	F	50	Y

cDNA numbering is based on reference database: The Polycystic Kidney Disease Mutation Database (PKDB server) [5].

HD - patients from dialysis centres in Czech Republic, F - female, M - male, HT - Hypertension, Y - yes, N - no;

Nonsense mutations in the *PKD1 *gene were the most frequent sequence changes in our patients. They were detected in 50.0% of affected individuals. Two short deletions in two patients (HD 2, HD 41) and one insertion/duplication (HD 12) were detected. Truncating mutations were found in 70.0% of cases (including in-frame deletion and putative splice site mutation), which is in concordance with previous reports using other methods of detection, such as DHPLC or direct sequencing [19].

The nonsense mutation p.R4021X was found in two unrelated Czech patients (fam. 281, HD 31). This nonsense mutation was frequently detected in other studies [19,27]. These C → T change in CpG dinucleotides may be recurrent. Recurrent mutations account for 30% of affected families and may simplify molecular diagnostics. The clinical course was quite severe in both Czech families. All family members from the hemodialyzed patient (HD 31), who had renal failure at the age of 36 years, were examined. Four of his siblings had renal failure at the ages of 54, 40, 43, and 42 years. Two affected sisters from family 281 had renal failure at the age of 50 years with manifestation and complications of the disease (such as severe hypertension, urolithiasis and recurrent cyst infections) from 30 years of age.

**Figure 3:**
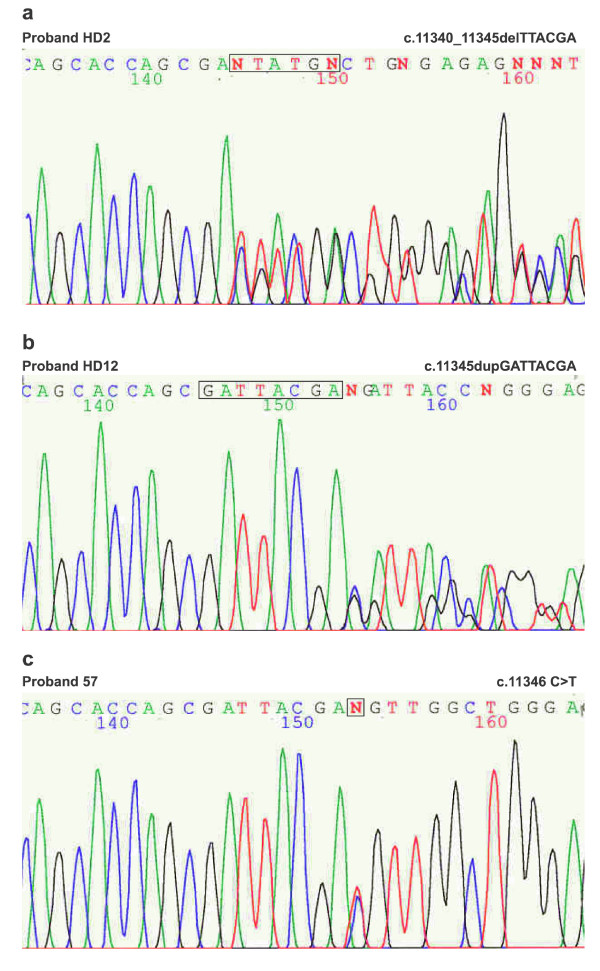
**The area of frequent mutations/polymorphisms in the *PKD1 *gene**. **a**. Sequencing pattern of the exon 40 (forward) in proband HD2 with heterozygous deletion of 6 bases in-frame c.11340_11345delTTACGA (p.Y3781_D3782del) of the *PKD1 *gene. **b**. Sequencing pattern of the exon 40 (forward) in proband HD12 with heterozygous duplication of 8 bases c.11345dupGATTACGA (p.D3782EfsX46) of the *PKD1 *gene. **c**. Sequencing pattern of the exon 40 (forward) in proband 57 with silent nucleotide change c.11346 C>T (p.A3782A) of the *PKD1 *gene.

A short deletion of 6 bases c.11340_11345delTTACGA is an in-frame putative mutation which ends in the absence of two amino acids p.Y3781_D3782del (Figure 3a). The proband already had ESRD at the age of 42 years. This deletion is in the area of frequent mutations/polymorphisms (Figure 3). The deletion was not present in the 27 year-old son with normal ultrasound. The deletion was present in the 29 year-old son who already suffered from mild renal insufficiency (serum creatinine 150 μmol/l). We would have predicted a milder clinical course in these two patients with ADPKD; however, both of them did not regularly use antihypertensive drugs and so did suffer from severe hypertension.

The deletion of 22 bases c.11623_11645del22 (p.A3875PfsX62) in exon 42 was detected in the patient with ESRD at the age of 44 years. This frameshift mutation leads to the formation of 62 new amino acids and then to a shorter protein.

The duplication of 8 bases c.11345dupGATTACGA (p.D3782EfsX46) (Figure 3b) in exon 40 was identified in the patient with ESRD at the age of 45 years. This change is also in the area of frequent mutations/polymorphisms (Figure 3). After 46 new amino acids, the protein is terminated.

The substitution in intron 36 (c.10821+1G → C) in the patient with ESRD at the age of 45 years (HD 15) was identified. This change is located just in the conserved donor splice site and will probably lead to an abnormal splicing. No mRNA experiment was available in our laboratory to confirm this hypothesis, but it has been well demonstrated in other genes. The effect of this substitution could be predicted either as exon skipping (in this case the skipping of exon 36) or frameshift [19,28]. This probable splice site mutation in intron 36 was also found in the 27 year-old son of the proband with enlarged cystic kidneys upon ultrasound, but was missing in two other sons of the proband (30 and 26 years-old) with normal ultrasounds. The causality of this substitution is thus highly probable. In addition, another substitution c.11080C → T (p.C3694R) in exon 38 was identified in the same family. The level of conservation of this missense variant is relatively low, though it is conserved well in mammals (Figure 2). Moreover, it segregates with the affected individuals in the family and is not present in 200 unrelated healthy controls. This missense variant in exon 38 can eventually contribute to the expansion of the severe clinical course of ADPDK as well and intensify the negative effect of probable splice site mutation in intron 36 on the function of polycystin-1. We can thus only speculate, which of these two substitutions is really causative.

Together with the potential missense mutation c.11080C → T (p.C3694R) in exon 38, six putative missense mutations in ADPKD patients (30.0% of all mutations) were detected. The most important factor in determining whether a missense change was likely pathogenic was the degree to which the corresponding sequence of amino acids was conserved. All missense variants segregated with affected members within a family. Moreover, 200 unrelated healthy individuals were tested and no similar changes were observed.

The putative missense mutation c.10850 T → C (p.L3617P) in exon 37 was identified in the family 340. This missense variant was present in the proband with enlarged cystic kidneys, in his son (multiple cysts at the age of 30 years) and in his daughter (two renal cysts at the age of 10 years). Leucine (a neutral hydrophobic amino acid) was changed to proline (which has an aliphatic side chain unlike other amino acids). Leucine in this position is conserved in all mammals, xenopus and trichoplax studied (Figure 2).

The putative missense mutation c.11078 C → T (S3693L; family 173) leads to the change of serine (a neutral hydrophilic amino acid) into leucine (a neutral hydrophobic amino acid). Serine is conserved only in mammals (Figure 2). This substitution was present in the proband (normal serum creatinine at the age of 38 years, enlarged cystic kidneys on ultrasound), in his mother (with ESRD at the age of 55 years, severe hypertension) and in his 7 year-old daughter (two renal cysts on ultrasound). This substitution was not present in the brother (normal ultrasound at the age of 40 years) and in the son (normal ultrasound at the age 12 year) of the proband.

The putative missense mutation c.11258 G → A (p.R3753Q) in exon 39 was identified in the family 329. Arginine (a basic amino acid) was changed to glutamine (a neutral amino acid containing an amino and an amide group). The grandfather of the proband had ESRD at the age of 59 years, the 58 year-old female suffered from moderate renal insufficiency (serum creatinine 220 μmol/l). The 32 year-old female was diagnosed by ultrasound at the age of 8 years and suffered from severe hypertension and from complicated renal infections. This putative missense mutation was described in another report [19].

The putative missense mutation c. 12648 A → C (p.Q4216H) in exon 46 was identified in the family 237. Glutamine (a neutral amino acid containing an amino and an amide group) was changed to histidine (a basic amino acid with an imidazole ring, which is frequently found in the reactive centre of proteins). Glutamine in this position was found in only a small portion of those mammals that have been compared (Figure 2). The grandfather of the female proband had ESRD at the age of 48 years; the mother of the proband started with hemodialysis at the age of 42 years. However, sequence analysis of this missense variant revealed that in some mammals, such as *Mus musculus *and *Rattus norvegicus*, histidine in position 4216 is conserved. So, it remains unclear whether this substitution is casual or a very rare sequence change without a pathological manifestation.

The putative missense mutation c. 12683 G → C (p.R4228P) in exon 46 was identified in the patient 38 from the hemodialysis center and is probably responsible for ADPKD. Arginine (a basic amino acid) was changed to proline (which has an aliphatic side chain unlike the other amino acids). Arginine in this position is conserved in a major part of those species that have been compared (Figure 2).

Polycystin-1 is a complicated transmembrane protein, whose structure has not been experimentally determined yet. Thus, all reports are based only on a theoretical model of polycystin-1. The model of UniProtKB/Swiss-Prot entry P98161 was used in our report [25].

Five mutations (p.R3753Q, p.Y3781_D3782del, p.D3782EfsX46, p.W3806X, and p.A3875PfsX62) were identified in the 202 amino acid long putative extracellular domain of polycystin-1. This extracellular domain is a part of the polycystin cation channel (PRO13122; AA3711-4113). This domain is supposed to be post-translationally modified by glycosylation. Additionally, the following putative cytoplasmatic domain, formed by 27 amino acids, is a part of the polycystin cation channel as well (PRO13122; AA3711-4113). Three mutations (p.Q4011X [29], p.L4019X, p.R4021X [27]) were detected in this intracellular domain of polycystin-1. The C terminus of polycystin-1 harbors a coiled-coil domain that is involved in the physical interaction with polycystin-2. Together, they form functional polycystin complexes *in vivo*. Three mutations (p.R4228P, p.Q4231X, and p.Q4242X) were identified in this coiled-coil domain.

## Conclusion

In conclusion, twenty putative mutations in the non-duplicated part of the *PKD1 *gene in 90 Czech individuals were detected. Fifteen of these putative mutations are novel (unique in Czech population). Truncated mutations, detected in 70% of the cases, were the most frequent mutations.

## Competing interests

The authors declare that they have no competing interests.

## Authors' contributions

JS carried out the molecular genetic studies, majority of mutation screening in *PKD1 *and participated in the design of the study. JR, MM, and OV collected clinical data and blood samples. SS carried out mutation screening in *PKD1*. VK carried out sequencing of *PKD1 *mutations. PL performed tests of occurence of misssense mutations in control set of individuals. MK conceived the study. All authors read and approved the final manuscript.

## Pre-publication history

The pre-publication history for this paper can be accessed here:



## References

[B1] Gabow PA (1991). Polycystic kidney disease: clues to pathogenesis. Kidney Int.

[B2] Gabow PA (1993). Autosomal dominant polycystic kidney disease. N Engl J Med.

[B3] European Polycystic Kidney Disease Consortium (1994). The polycystic kidney disease 1 gene encodes a 14 kb transcript and lies within a duplicated region on chromosome 16. Cell.

[B4] International Polycystic Kidney Disease Consortium Polycystic kidney disease (1995). The complete structure of the PKD1 gene and its protein. Cell.

[B5] The Polycysic Kidney Disease Mutation Database. http://pkdb.mayo.edu/.

[B6] Mochizuki T, Wu G, Hayashi T, Xenophontos SL, Veldhuisen B, Saris JJ, Reynolds DM, Cai Y, Gabow PA, Pierides A, Kimberling WJ, Breuning MH, Deltas CC, Peters DJ, Somlo S (1996). PKD2, a gene for polycystic kidney disease that encodes an integral membrane protein. Science.

[B7] Daoust MC, Reynolds DM, Bichet DG, Somlo S (1995). Evidence for a third genetic locus for autosomal dominant polycystic kidney disease. Genomics.

[B8] Torra R, Badenas C, Darnell A, Nicolau C, Volpini V, Revert L, Estivill X (1996). Linkage, clinical features, and prognosis of autosomal dominant polycystic kidney disease types 1 and 2. J Am Soc Nephrol.

[B9] Hateboer A, van Dijk MA, Bogdanova A, Coto E, Saggar-Malik KA, San Millan JL, Torra R, Breuning M, Ravine D (1999). Comparison of phenotypes of polycystic kidney disease types 1 and 2. Lancet.

[B10] Torres VE, Harris PC (2006). Mechanisms of disease: Autosomal dominant and recessive polycystic kidney disease. Nat Clin Pract Nephrol.

[B11] Qian F, Germino FJ, Cai Y, Zhang X, Somlo S, Germino GG (1997). PKD1 interacts with PKD2 through a probable coiled coil domain. Nat Genet.

[B12] Tsiokas L, Kim E, Arnould T, Sukhatme UP, Walz G (1997). Homo- and heterodimeric interactions between the gene products of PKD1 and PKD2. Proc Natl Acad Sci USA.

[B13] Yoder BK, Hou X, Guay-Woodford LM (2002). The polycystic kidney disease proteins, polycystin-1, polycystin-2, polaris, and cystin, are co-localized in renal cilia. J Am Soc Nephrol.

[B14] Nauli SM, Alenghat FJ, Luo Y, Williams E, Vassilev P, Li X, Elia AE, Lu W, Brown EM, Quinn SJ, Ingber DE, Zhou J (2003). Polycystins 1 and 2 mediate mechanosensation in the primary cilium of kidney cells. Nat Genet.

[B15] Rossetti S, Burton S, Strmecki L, Pon GR, San Millan JL, Zerres K, Barratt TM, Ozen S, Torres VE, Bergstrahl EJ, Winearls CG, Harris PC (2002). The position of the polycystic kidney disease 1 (PKD1) gene mutation correlates with the severity of renal disease. J Am Soc Nephrol.

[B16] Watnick T, Phakdeekitcharoen B, Johnson A (1999). Mutation detection of PKD1 identifies a novel mutation common to three families with aneurysms and/or very-early-onset disease. Am J Hum Genet.

[B17] Peral B, Ong ACM, San Millan JL, Gamble V, Rees L, Harris PC (1996). A stable, nonsense mutation associated with a case of infantile onset polycystic kidney disease 1 (PKD1). Hum Mol Genet.

[B18] Hateboer A, Veldhuisen B, Peters DJM, Breuning MH (2000). Location of mutations within the PKD2 gene influences clinical outcome. Kidney Int.

[B19] Rossetti S, Consugar MB, Chapman AB, Torres VE, Guay-Woodford LM (2007). Comprehensive molecular diagnostics in autosomal dominant polycystic kidney disease. J Am Soc Nephrol.

[B20] Ravine D, Gibson RN, Walker RG, Sheffield LJ, Kincaid-Smith P, Danks DM (1994). Evaluation of ultrasonographic diagnostic criteria for autosomal dominant polycystic kidney disease 1. Lancet.

[B21] Miler SA, Dykes DD, Polesky MF (1988). A simple salting out procedure for extracting DNA from human nucleated cells. Nucl AcidRes.

[B22] Perrichot RA, Mercier B, Simom PM, Whebe B, Cledes J, Ferec C (1999). DGGE screening of PKD1 gene reveals novel mutations in a large kohort of 146 unrelated patiens. Hum Genet.

[B23] National Center for Biotechnology Information. BLAST. http://www.ncbi.nlm.nih.gov/blast/.

[B24] BioEdit. Biological sequence alignment editor for Windows 95/98/NT/2000/XP. http://www.mbio.ncsu.edu/BioEdit/BioEdit.html.

[B25] UniProtKB/Swiss-Prot entry P98161. P98161.

[B26] Takahashi N, Hiyami K, Kodaira M, Satoh C (1990). An improved method for the detection of genetic variations in DNA with denaturating gradient electrophoresis. Mut Res.

[B27] Turco AE, Rossetti S, Bresin E, Corrà S, Restagno G, Carbonara A, De Prisco O, Gammaro L, Maschio G, Pignatti PF (1996). Detection of two different nonsense mutations in exon 44 of the PKD1 gene in two unrelated Italian families with severe autosomal dominant polycystic kidney disease. Nephrol Dial Transplant.

[B28] Neklasov DW, Solomon CH, Dalton AL, Kuwada SK, Burt RW (2004). Intron 4 mutation in APC gene results in splice defect and attenuated FAPphenotype. Familial Cancer.

[B29] Daniells C, Maheshwar M, Lazarou L, Davies F, Coles G, Ravine D (1998). Novel and recurrent mutations in the PKD1 gene. Hum Genet.

[B30] The Human Gene Mutation Database at the Institute of medical Genetics in Cardiff. http://www.hgmd.cf.ac.uk/ac/index.php.

[B31] Rossetti S, Strmecki L, Gamble V, Burton S, Sneddon V, Peral B, Roy S, Bakkaloglu A, Komel R, Winearls CG, Harris PC (2001). Mutation analysis of the entire PKD1 gene: genetic and diagnostic implications. Am J Hum Genet.

[B32] Peral B, Gamble V, Strong C, Ong AC, Sloane-Stanley J, Zerres K, Winearls CG, Harris PC (1997). Identification of mutatio in the duplicated region of the PKD1 gene by a novel approach. Am J Hum Genet.

[B33] Aguiari G, Savelli S, Garbo M, Bozza A, Augello G, Penolazzi L, De Paoli Vitali E, La Torre C, Cappelli G, Piva R, del Senno L (2000). Novel splicing and missense mutations in autosomal dominant polycystic kidney disease 1 (PKD1) gene: expression of mutated genes. Hum Mutat.

[B34] Rossetti S, Chauveau D, Walker D, Saggar-Malik A, Winearls CG, Torres VE, Harris PC (2002). A complete mutation screen of the ADPKD genes by DHPLC. Kidney Int.

[B35] Perrichot R, Mercier B, Carre A, Cledes J, Ferec C (2000). Identification of 3 novel mutations (Y4236X, Q3820X, 11745+2 ins3) in autosomal dominant polycystic kidney disease 1 gene (PKD1). Hum Mutat.

[B36] Rosseti S, Bresin E, Restagno G, Carbonara A, Corrà S, De Prisco O, Pignatti PF, Turco AE (1996). Autosomal dominant polycystc kidney disaese (ADPKD) in an Italian family carrying a novel nonsense mutation and two missense changes in exons 44 and 45 of the PKD1 gene. Am J Med Genet.

[B37] Vouk K, Strmecki L, Stekrova J, Reiterova J, Bidovec M, Hudler P, Kenig A, Jereb S, Zupanic-Pajnic I, Balazic J, Haarpaintner G, Leskovar B, Adamlje A, Skoflic A, Dovc R, Hojs R, Komel R (2006). PKD1 and PKD2 mutations in Slovenian families with autosomal dominant polycystic kidney disease. BMC Med Genet.

[B38] Garcia-Gonzalez MA, Jones JG, Allen SK, Palatucci CM, Batish SD, Seltzer WK, Lan Z, Allen E, Qian F, Lens XM, Pei Y, Germino GG, Watnick TJ (2007). Evaluating the clinical utility of a molecular genetic test for polycystic kidney disease. Mol Genet Metab.

[B39] Badenas C, Torra R, San Millán JL, Lucero L, Milà M, Estivill X, Darnell A (1999). Mutational analysis within the 3'region of the PKD1 gene. Kidney International.

[B40] Peral B, San Millán JL, Ong AC, Gamble V, Ward CJ, Strong C, Harris PC (1996). Screening the 3' region of the polycystic kidney disease 1 (PKD1) gene reveals six novel mutations. Am J Hum Genet.

